# Expressions of ECE-CYC2 clade genes relating to abortion of both dorsal and ventral stamens in *Opithandra *(Gesneriaceae)

**DOI:** 10.1186/1471-2148-9-244

**Published:** 2009-10-07

**Authors:** Chun-Feng Song, Qi-Bing Lin, Rong-Hua Liang, Yin-Zheng Wang

**Affiliations:** 1 State Key Laboratory of Systematic and Evolutionary Botany, Institute of Botany, Chinese Academy of Sciences, 20 Nanxincun, Xiangshan, Beijing 100093, PR China

## Abstract

**Background:**

ECE-CYC2 clade genes known in patterning floral dorsoventral asymmetry (zygomorphy) in *Antirrhinum majus *are conserved in the dorsal identity function including arresting the dorsal stamen. However, it remains uncertain whether the same mechanism underlies abortion of the ventral stamens, an important morphological trait related to evolution and diversification of zygomorphy in Lamiales *sensu lato*, a major clade of predominantly zygomorphically flowered angiosperms. *Opithandra *(Gesneriaceae) is of particular interests in addressing this question as it is in the base of Lamiales *s.l*., an early representative of this type zygomorphy.

**Results:**

We investigated the expression patterns of four ECE-CYC2 clade genes and two putative target *cyclinD3 *genes in *Opithandra *using RNA *in situ *hybridization and RT-PCR. *OpdCYC *gene expressions were correlated with abortion of both dorsal and ventral stamens in *Opithandra*, strengthened by the negatively correlated expression of their putative target *OpdcyclinD3 *genes. The complement of *OpdcyclinD3 *to *OpdCYC *expressions further indicated that *OpdCYC *expressions were related to the dorsal and ventral stamen abortion through negative effects on *OpdcyclinD3 *genes.

**Conclusion:**

These results suggest that ECE-CYC2 clade TCP genes are not only functionally conserved in the dorsal stamen repression, but also involved in arresting ventral stamens, a genetic mechanism underlying the establishment of zygomorphy with abortion of both the dorsal and ventral stamens evolved in angiosperms, especially within Lamiales *s.l*.

## Background

One important event during the evolution of angiosperms is the emergence of flower bilateral symmetry, i.e. zygomorphy, a key innovation associated with important adaptive radiations [1]. Several zygomorphic clades have independently evolved successfully from actinomorphic ancestors in angiosperms, including Lamiales *sensu lato *that includes a major genetic model organism snapdragon (*Antirrhinum majus*) [2,3].

In *A*.*majus*, *CYCLOIDEA *(*CYC*) and *DICHOTOMA *(*DICH*) are essential for the development of dorsoventral asymmetry in flowers due to their dorsal identity function, i.e. controlling the fate of the dorsal floral organs in the second and third whorls [4,5]. *CYC *promotes cell expansion in the dorsal petals, while it arrests the growth of the dorsal stamen to become a staminode [4,5]. Meanwhile, *DICH *activity affects the internal asymmetry of the dorsal petals [4,5]. The ability of *CYC *to arrest the dorsal stamen depends on its negative effect on expression of cell-cycle genes, such as *cyclin D3b *[3,6]. *CYC *and *DICH *encode proteins within the ECE-CYC2 clade (ECE lineage, CYC/TB1 subfamily) in the TCP family of transcription factors with TCP domain related to cell proliferation [3,7-12]. In legumes, distantly related to *A. majus*, several *CYC *homologues, such as *LjCYC*2 in *Lotus *and *PsCYC2 *in pea, also have the function in establishing dorsal identity in legume flowers [13,14]. In *Arabidopsis thaliana*, a model eudicot species with ancestrally actinomorphic flowers, and its close relative *Iberis amara*, *CYC *homologues, *TCP1 *and *IaTCP1 *genes are characteristic of dorsal identity function, in which *IaTCP1 *dorsal-specific expression represses the two dorsal petal development in *Iberis amara *[15,16]. Recent studies in the sunflower family (Asteraceae) show that *CYC*-like genes, i.e. *RAY1*, *RAY2 *in *Senecio *and *GhCYC2 *in *Gerbera*, have played a key role in the establishment and evolution of the capitulate inflorescence [17,18]. Therefore, it is suggested that *CYC*-like TCP genes have been recruited multiple times for a role of dorsal identity and its modifications in establishing zygomorphy in core eudicots [3,19]. Even though the genetic control for the floral dorsoventral asymmetry has been intensively studied in model systems, it is still a great challenge to explain how modifications of development led to the transformation among different types of zygomorphy and the morphological diversification of zygomorphy in angiosperms, especially in Lamiales *s.l*., a major clade of predominantly zygomorphically flowered angiosperms.

Zygomorphy is believed to be ancestral in Lamiales *s.l*. [2,19,20]. Most zygomorphic groups in Lamiales *s.l*. have a pentamerous perianth with four stamens plus a dorsal staminode and two carpels as in *A*.*majus*. However, there is a great variation in morphology and number of corolla lobes and stamens [1]. The dorsal staminode can be completely lost as in *Rehmannia *and *Veronica *(Scrophulariaceae *sensu lato*) [2,21,22] and the two lateral stamens may become aborted instead of one dorsal staminode as in *Mohavea *(Scrophulariaceae *s.l*.) and *Chirita *(Gesneriaceae) [23-25]. In some cases, the two ventral stamens may become staminodes rather than the lateral stamens and the dorsal one, such as in *Opithandra *and *Epithema *(Gesneriaceae) [24]. In extreme cases, each flower may have only a single stamen as in *Hippuris *(Scrophulariaceae *s.l*.) [20]. In *Mohavea*, a close relative of *A. majus*, there is a derived floral morphology with abortion of both the dorsal and lateral stamens unlike the flowers of *A*.*majus *with abortion of only the dorsal stamen [23]. The derived floral morphology of *Mohavea *is correlated with the expression changes of *McCYC/McDICH *via *CYC/DICH*, i.e. expansion from the dorsal to both the dorsal and lateral stamens [23]. A similar correlation of expanded expression of *CYC*-like genes with abortion of both the dorsal and lateral stamens is also observed in *Chirita *(Gesneriaceae) [25]. However, we are still not clear about the abortion of the ventral stamens that has been involved in the evolutionary shifts of stamen number during the morphological diversification of zygomorphy in Lamiales *s.l*. [1].

In addition, in the *backpetals *mutant in *A*.*majus*, the ectopic expression of *CYC *in the lateral and ventral positions results in a dorsalized corolla. However, it seems likely that the androecial development is not affected by the ectopic expression of *CYC *because the two lateral and two ventral stamens are still fertile [5,26]. In the actinomorphic flower of legume *Cadia*, no *LegCYC1B *mRNA is detected in stamens [27]. It is hard to determine whether *LegCYC1B *or other *CYC *homologues in legumes have a role in controlling androecial development from data to date [13,14,21]. *RAY2 *in *Senecio *and *GhCYC2 *in *Gerbera *(Asteraceae) mainly promote the growth of the ligule (the ventral petals) in ray florets and are excluded from the dorsal rudimentary petals [17,18]. In *Veronica *and *Gratiola *(Scrophulariaceae *s.l*.), some of *CYC*-like genes have dorsal-specific expressions while some have lost this feature, but their expressions do not correlate with ventral stamen arrest [21]. Therefore, expression data correlated with the ventral stamen abortion have not been reported yet for members of the ECE-CYC2 clade. It is uncertain whether abortion of the ventral stamens is related to *CYC*-like gene activities or to the effect of an unknown analogous counterpart of *CYC*-like genes, such as members of ECE-CYC3 clade or other factors [3,12,20].

**Figure 1:**
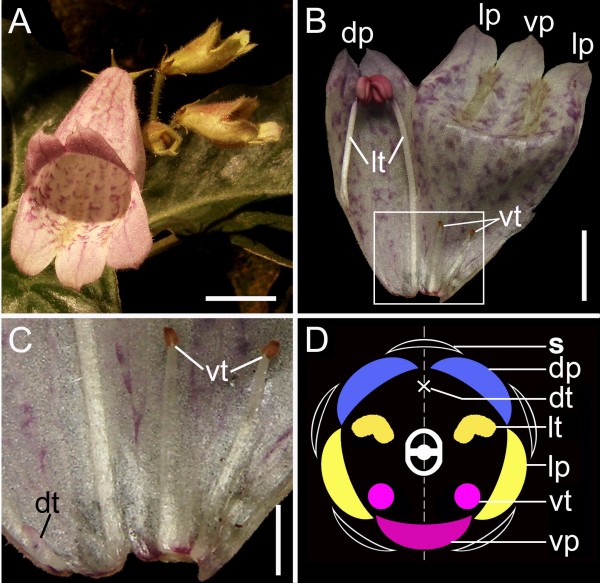
**Flower morphology of *Opithandra dinghushanensis***. **A) **Inflorescence with flowers near or at anthesis, showing strongly zygomorphic corolla; **B) **An opened corolla at anthesis showing two dorsal petals smaller than two lateral and one ventral petals, and androecium with two fertile lateral stamens and two ventral and one dorsal staminodes; **C) **Magnification of the framed part in (B), showing two infertile lateral stamens with short filaments and small sterile anthers, and a tiny dorsal staminode that is barely visible; **D) **Floral diagram; Scale bars, 10 mm (A), 7 mm (B) and 3 mm (C). dp, dorsal petal (in blue); dt, dorsal staminode; lp, lateral petal (in yellow); lt, lateral stamen (in yellow); s, sepal; vp, ventral petal (in pink); vt, ventral staminode (in pink).

The family Gesneriaceae is sister to the remainder of Lamiales *s.l*. [28] and has diverse forms of zygomorphy relating to the floral organ differentiation early in the order [1,2,24]. In Gesneriaceae, *Opithandra *exhibits a peculiar floral morphology, where only the two lateral stamens are fertile and both the dorsal and ventral stamens are aborted in the third whorl (Figure 1). Phylogenetic analyses suggest that the floral morphology of *Opithandra *is likely derived from a weakly zygomorphic flower with four fertile stamens and a dorsal staminode [24]. Therefore, *Opithandra *represents an ideal candidate for exploring a potentially novel genetic mechanism underlying the establishment of zygomorphy with ventral stamen arrest in angiosperms, especially in Lamiales *s.l*.

Here we report that there is a correlation between *OpdCYC *gene expressions and abortion of both the dorsal and ventral stamens in *Opithandra*, strengthened by the negatively correlated expression of their putative direct target *OpdcyclinD3 *genes. The novel patterns of *CYC*-like gene expressions in *Opithandra *indicate that ECE-CYC2 clade TCP genes are involved in the ventral stamen repression evolved within Lamiales *s. l*..

## Results

### Sequence and phylogenetic analyses of OpdCYC and OpdcyclinD3

**Figure 2:**
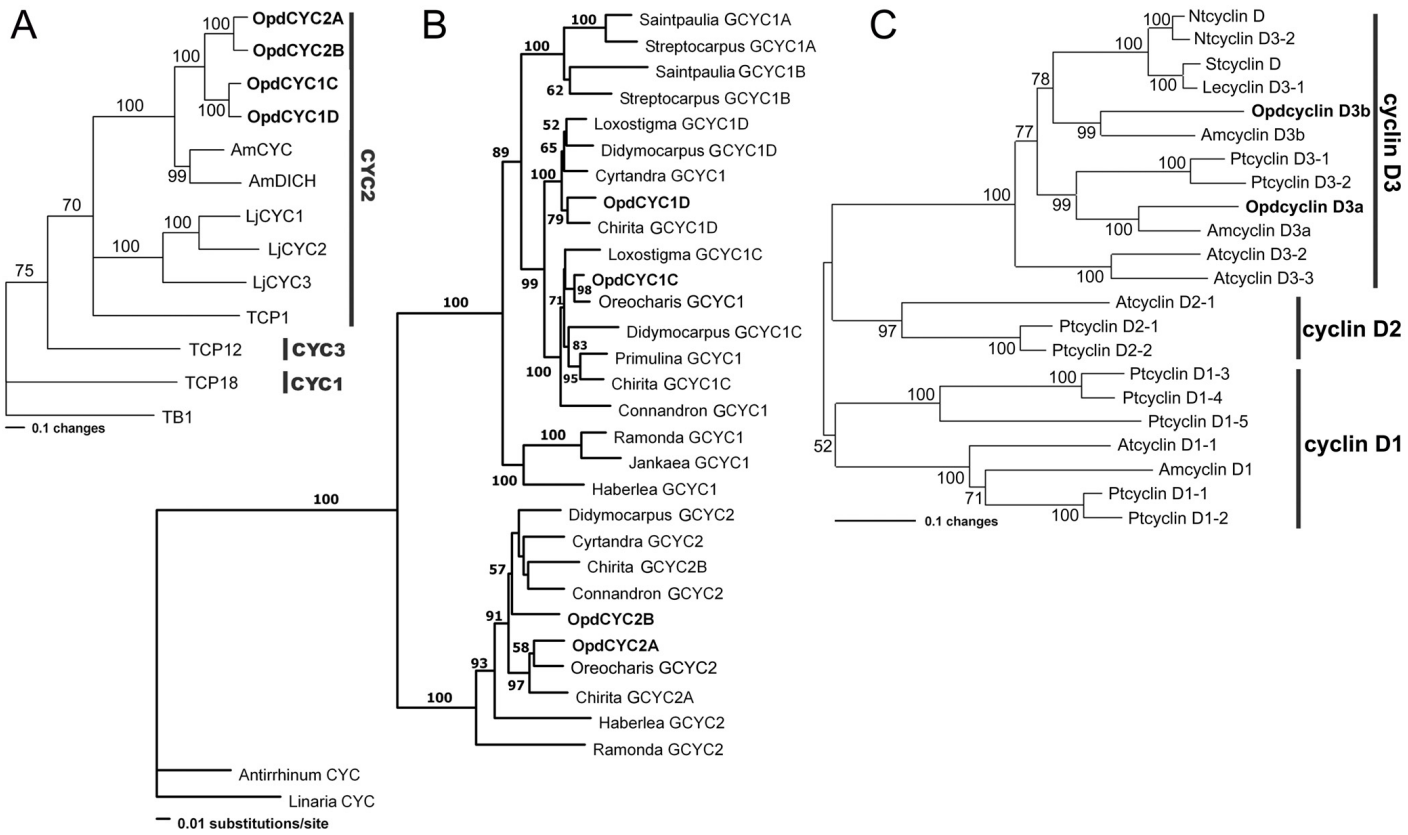
**Neighbor-joining trees of proteins encoded by *CYC*-like and D-type *cyclin *genes**. **A) **Neighbor-joining tree of proteins encoded by the ECE lineage genes in CYC/TB1 subfamily, showing that OpdCYC1C/1D and OpdCYC2A/2B form a branch that is sister to AmCYC/AmDICH from *Antirrhinum majus*, which belong to the CYC2 clade in the ECE lineage. **B) **Phylogram of GCYC, showing the phylogenetic relations of *OpdCYC *genes with other GCYC in Gesneriaceae. **C) **Neighbor-joining tree of proteins encoded by D-type *cyclin *genes, showing that OpdcyclinD3a and OpdcyclinD3b are clustered with cyclinD3a and cyclinD3b clades, respectively, in the cyclinD3 lineage. For sequence information see Methods. Phylogenetic analyses were conducted using PAUP*4.0b4a, and bootsrap values over 50% (1,000 replicates) are indicated for each branch.

We isolated four *CYC*-like genes from *Opithandra dinghushanensis*, named *OpdCYC1C*, *OpdCYC1D*, *OpdCYC2A *and *OpdCYC2B*. The full length open reading frames (ORFs) of *OpdCYC1C*, *OpdCYC1D*, *OpdCYC2A *and *OpdCYC2B *are 1017 base pair (bp), 1038 bp, 1044 bp and 993 bp, respectively (Additional file 1A). Sequence analyses show that they share 43-48% and 45-51% identity with *AmCYC *at nucleotide and amino acid levels, respectively. When comparing the TCP and R domains, they share 90-95% identity with *AmCYC *at the amino acid level, suggesting they are functionally related. Phylogenetic analyses show that *OpdCYC *genes have a close relationship with *AmCYC *and *AmDICH*, and, along with *AmCYC *and *TCP1*, belong to the ECE-CYC2 clade in the ECE lineage (CYC/TB1 subfamily) of TCP gene family [12] (Figure 2A) (We have not found a member of the ECE-CYC3 clade yet in Gesneriaceae, probably failed to amplify them because of difficulty in designing specific primers for this clade). *OpdCYC *genes are closely related to *GCYC *from *Oreocharis *among *GCYC *genes in Gesneriaceae (Figure 2B).

Two D3-type *cyclin *genes, designated as *OpdcyclinD3a *and *OpdcyclinD3b*, were isolated from *O. dinghushanensis *with full length ORFs of 1563 bp and 1200 bp, respectively (Additional file 1B). The two D3-type *cyclin *genes contain a cyclin box [29] and the putative (Rb)-binding motif (*L*x*C*x*E*, where x is any amino acid) which are found both in animals [30,31] and plants [6,32,33]. Phylogenetic analyses show that *OpdcyclinD3 *genes belong to cyclinD3a and cyclinD3b clade, respectively, in the cyclinD3 lineages, in which they have close relations with AmcyclinD3a and AmcyclinD3b from *A*.*majus*, respectively (Figure 2C).

### Gene mRNA expression patterns

**Figure 3:**
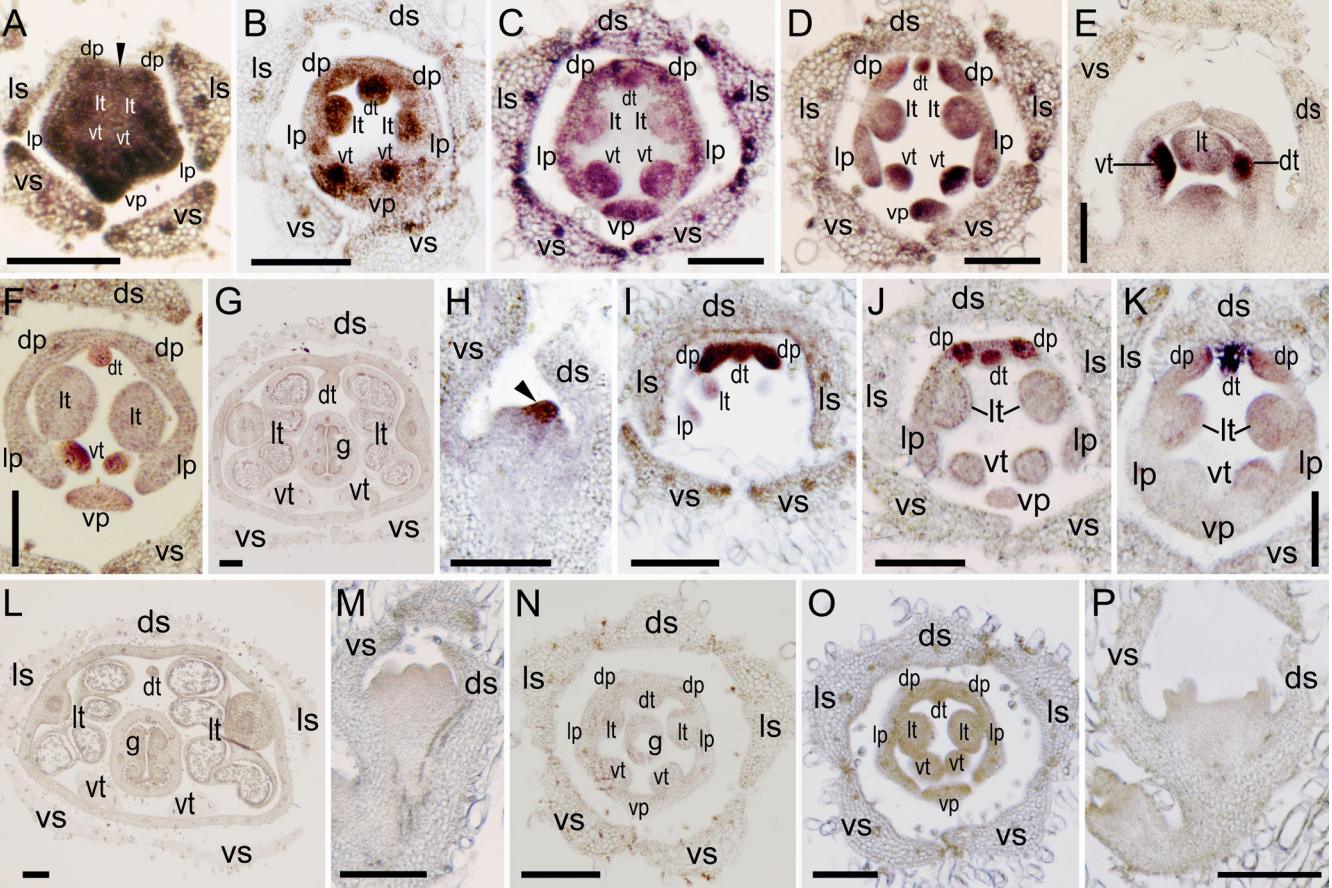
**Tissue-specific expression of *OpdCYC1 *and *OpdCYC2 *during floral development in *Opithandra dinghushanensis***. **A-G) **RNA *in situ *hybridizations with antisense probe of *OpdCYC1C*. **A) **Its mRNA is first detected in all five petal and stamen primordia with weak signals in lateral edges and vascular tissue of sepals. **B-D) **Its mRNA then weakened in two lateral stamens but strong in both dorsal and ventral staminodes with weak mRNA in the ring meristem of corolla-tube. **C-D) **Successive sections from the same individual flower across base (C) and upper parts (D) of stamens. **E-F) **Its expression shifts to peripheries and becomes undetectable in two enlarged lateral stamens while remains strong in dorsal and ventral staminodes. **G) **Its mRNA is undetectable in stamens as pollen sacs begin development. **H-L) **RNA *in situ *hybridizations with antisense probe of *OpdCYC2A*. **H) **Its dense transcript accumulation first restricted to the dorsal side of the floral apex (arrow). **I-K) **Its strong expression then restricted to two dorsal petals and the dorsal staminode as they are initiated. Note its mRNA later becomes restricted to the dorsal-most parts in two dorsal petals (K). **L) **Its mRNA is undetectable in stamens as pollen sacs begin development. *OpdCYC1D *(M) and *OpdCYC2B *(N) mRNA is not detected in floral tissues. As a negative control, RNA *in situ *hybridizations with sense probes of *OpdCYC1C *(O) and *OpdCYC2A *(P) detect no signal in floral tissues. dp, dorsal petal; ds, dorsal sepal; dt, dorsal staminode; g, gynoecium; lp, lateral petal; ls, lateral sepal; lt, lateral stamen; vp, ventral petal; vs, ventral sepal; vt, ventral staminode. Scale bars, 150 μm.

**Figure 4:**
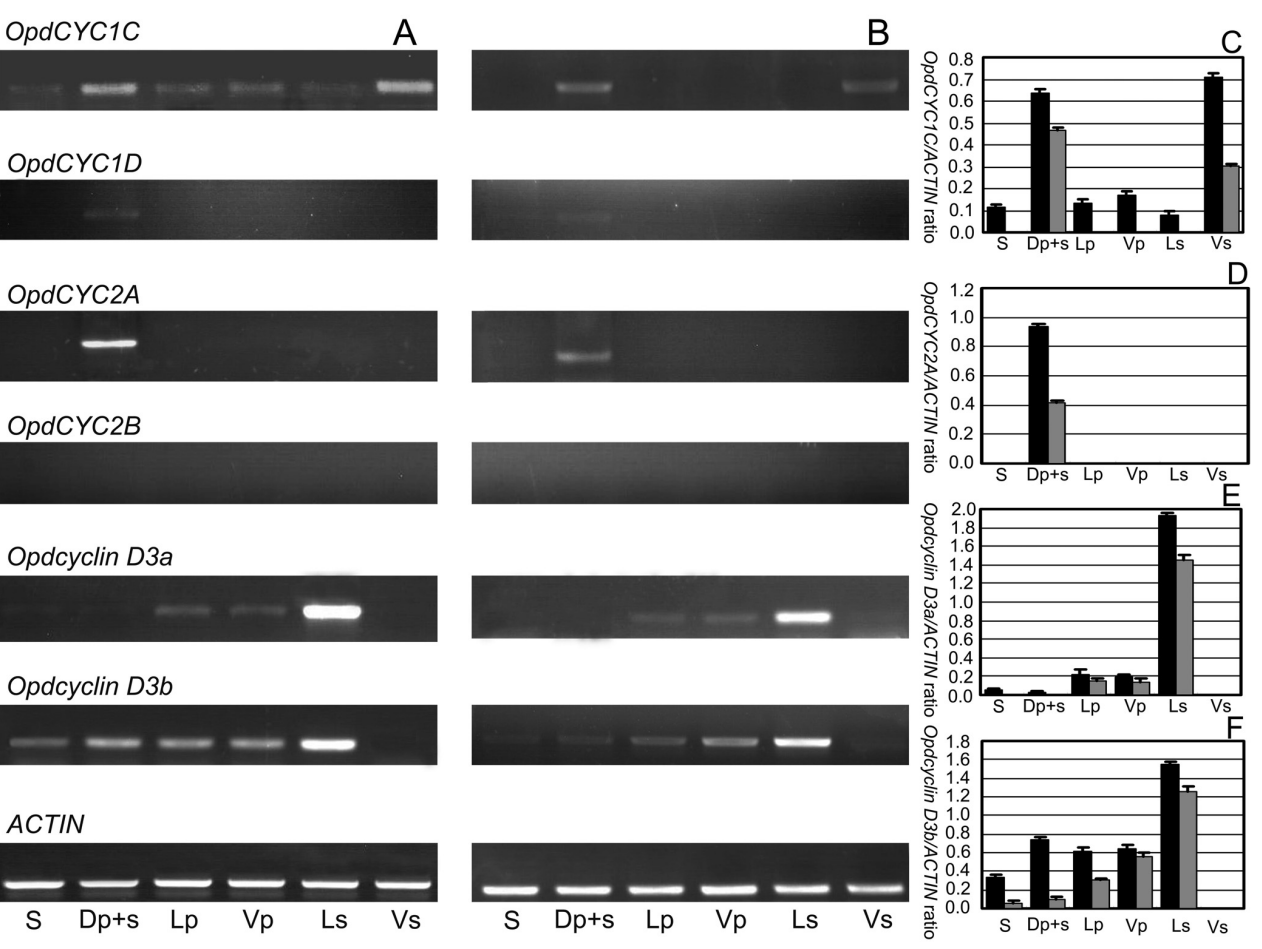
**Gene-specific semiquantitative RT-PCR on RNA prepared from dissected *Opithandra dinghushanensis *flowers**. **A) **sepal (S), dorsal petal+staminode (Dp+s), lateral/ventral petals (Lp/Vp), lateral stamens (Ls) and ventral staminode (Vs) were dissected from flower buds of middle-stage (less than 1 cm long). **B) **sepal (S), dorsal petal+staminode (Dp+s), lateral/ventral petals (Lp/Vp), lateral stamens (Ls) and ventral staminode (Vs) were dissected from flowers of late-stage (3-4 cm long). **C) **Relative level of *OpdCYC1C *mRNA expression in middle-stage (black) *vs*. late-stage (grey) compared with *ACTIN*. **D) **Relative level of *OpdCYC2A *mRNA expression in middle-stage (black) *vs*. late-stage (grey) compared with *ACTIN*. **E) **Relative level of *Opdcyclin D3a *mRNA expression in middle-stage (black) *vs*. late-stage (grey) compared with *ACTIN*. **F) **Relative level of *Opdcyclin D3b *mRNA expression in middle-stage (black) *vs*. late-stage (grey) compared with *ACTIN. ACTIN *was used for RT template control. The values (means ± SD) shown are determined from five independent experiments.

To assess the potential role of *CYC*-like genes in floral development, we conducted *in situ *hybridization complemented by gene-specific RT-PCR on *O. dinghushanensis*. As petal and stamen primordia began to emerge, *OpdCYC1C *mRNA was detected in all five petal and stamen primordia (Figure 3A). Weak mRNA signals were also detectable in the lateral edges and vascular tissue of sepals (Figure 3A). After primordial initiation of petals and stamens, *OpdCYC1C *expression signals were gradually weakened in the two lateral stamens (Figure 3B-D) with weak mRNA detected in the ring meristem of the corolla-tube outside the stamen primordia (Figure 3B-C). Figures 3C and 3D were the successive sections from the same individual flower across the base of stamen primordia (3C) and over their upper parts (3D), respectively, which showed a size reduction from the base to the upper part of the dorsal and ventral stamens. The mRNA signal of *OpdCYC1C *was weak in lateral stamens while strong in both dorsal and ventral staminodes in which its mRNA signal was stronger in the upper part than at the base (Figure 3C-D). *OpdCYC1C *mRNA also accumulated less in lateral petals than in dorsal and ventral petals (Figure 3D). As the lateral stamens enlarged laterally, weak *OpdCYC1C *mRNA shifted to peripheries and gradually became undetectable in the two lateral stamens, while *OpdCYC1C *transcripts continued to accumulate to a high level in the dorsal and ventral staminodes (Figure 3E-F). Meanwhile, *OpdCYC1C *mRNA became weak and not easily detectable in petals (Figure 3E-F). Weak mRNA signals of *OpdCYC1C *were also detected in the gynoecial primordium (Figure 3E). In the middle developmental stages with flower buds about 8 mm long, as stamens began filament elongation and anther differentiation, *OpdCYC1C *was strongly expressed in the dorsal region (the dorsal petals and staminode) and ventral staminode shown in RT-PCR while its weak mRNA signal was detected in sepals, lateral and ventral petals, and lateral stamens, in which the signal was much weaker in sepals and lateral stamens (Figure 4A, C). In the late stages, *OpdCYC1C *transcripts declined in the dorsal region and ventral staminodes, and were undetectable in other regions (Figure 4B-C). The *OpdCYC1C *mRNA was undetectable in stamens as pollen sacs began development in the two lateral anthers while the two ventral anthers became sterile (Figure 3G).

In contrast to *OpdCYC1C*, *OpdCYC2A *mRNA densely accumulated in the dorsal region of the floral apex as petals and stamens became visible (Figure 3H). Then, the *OpdCYC2A *expression signal was specifically concentrated in the dorsal petals and the dorsal staminode (Figure 3I-J). As floral organs developed, *OpdCYC2A *transcripts continued to be highly concentrated at the dorsal staminode and the dorsal-most parts of the two dorsal petals (Figure 3J-K). In the middle stages, the strong mRNA signal of *OpdCYC2A *was detected in the dorsal region (the dorsal petals and staminode) shown in RT-PCR that declined in late stages with no signal in other regions (Figure 4A-B, D). Its mRNA was undetectable in stamens as pollen sacs began development in the two lateral anthers while the two ventral anthers became sterile (Figure 3L). Even though *OpdCYC1D *expression was undetectable in floral tissues using *in situ *hybridization (Figure 3M), its very weak mRNA signals were detected in the dorsal region (the dorsal petals and staminode) from middle to late stages using RT-PCR (Figure 4A-B). *OpdCYC2B *mRNA was not detectable in floral tissues both using *in situ *hybridization and RT-PCR (Figure 3N, Figure 4A-B). No signal was detected in floral tissues with sense probes of *OpdCYC1C *(Figure 3O) and *OpdCYC2A *(Figure 3P).

To further elucidate the role of *OpdCYC *genes in floral development of *Opithandra dinghushanensis*, we carried out semiquantitative RT-PCR studies of two *OpdcyclinD3 *genes because *cyclinD3 *genes were previously revealed to be negatively controlled by *CYC *as shown in the mid-to-late stage flowers in the model organism snapdragon [6]. RT-PCR results showed that *OpdcyclinD3a *was strongly expressed in lateral stamens from middle to late stages while its weak mRNA signal was also detected in lateral and ventral petals (Figure 4A-B, E). *OpdcyclinD3a *mRNA was not detected either in the dorsal region (the dorsal petals and staminode) and ventral staminodes (Figure 4A-B, E). *OpdcyclinD3b *transcripts were widely distributed in floral tissues except ventral staminodes, in which its mRNA signal was strong in lateral stamens from middle to late stages (Figure 4A-B, F). Transcripts of *OpdcyclinD3b *detected in the dorsal region were likely mainly distributed in the dorsal petals (*OpdcyclinD3b *mRNA was uneasily detectable in the dorsal staminode using *in situ *hybridization (data not shown)).

## Discussion

The androecium of *Opithandra *only has two fertile stamens at the lateral positions with three sterile stamens (staminodes) at the dorsal and ventral sides (Figure 1) (also see [24]). The dorsal aborted stamen is tiny and barely detectable at anthesis while the two infertile ventral stamens have short filaments with very small and sterile anthers (Figure 1B-C). Correlative with the differentiation along the dorsoventral axis of the morphologically peculiar androecium, the *OpdCYC2A *strong expression is restricted to the dorsal staminode while *OpdCYC1C *transcripts are initially distributed in all five stamen primordia but later are concentrated in the dorsal and ventral staminodes to late stages. In the ECE lineage of CYC/TB1 subfamily, the TCP proteins in ECE-CYC2 clade studied to date function as negative regulators in stamen development, whereas they appear to vary in petal development according to the trait concerned [3-5,9,10,12,17,23,25,34,35]. The abortion of the dorsal stamen in *Antirrhinum *comes from *CYC *and *DICH *activities [4,5]. The *CYC*-like gene expression expansion from the dorsal to both the dorsal and lateral stamens is correlated with abortion of both the dorsal and lateral stamens in *Mohavea *and *Chirita *[23,25]. *TB1 *gene exhibits a mix feature of ECE-CYC1 and ECE-CYC2 clades and functions to suppress axillary meristem (CYC1) while retard stamen growth (CYC2) in maize [3,12,34]. In Asteraceae, a *CYC *homologue *GhCYC2 *from *Gerbera *functions by disrupting stamen development [17]. Given that *CYC*-like gene (ECE-CYC2 clade) function is conserved in repressing stamen development, *OpdCYC2A *expression restricted to the dorsal stamen and *OpdCYC1C *expression later concentrated in the dorsal and ventral stamens in the third whorl might be related to abortion of the dorsal and ventral stamens in *Opithandra*. In fact, the successive sections from the same individual flower indicate that correlative with *OpdCYC *gene strong expression, the early primordial growth have already been retarded in the dorsal and ventral staminodes in comparison with that in the lateral stamens.

**Figure 5:**
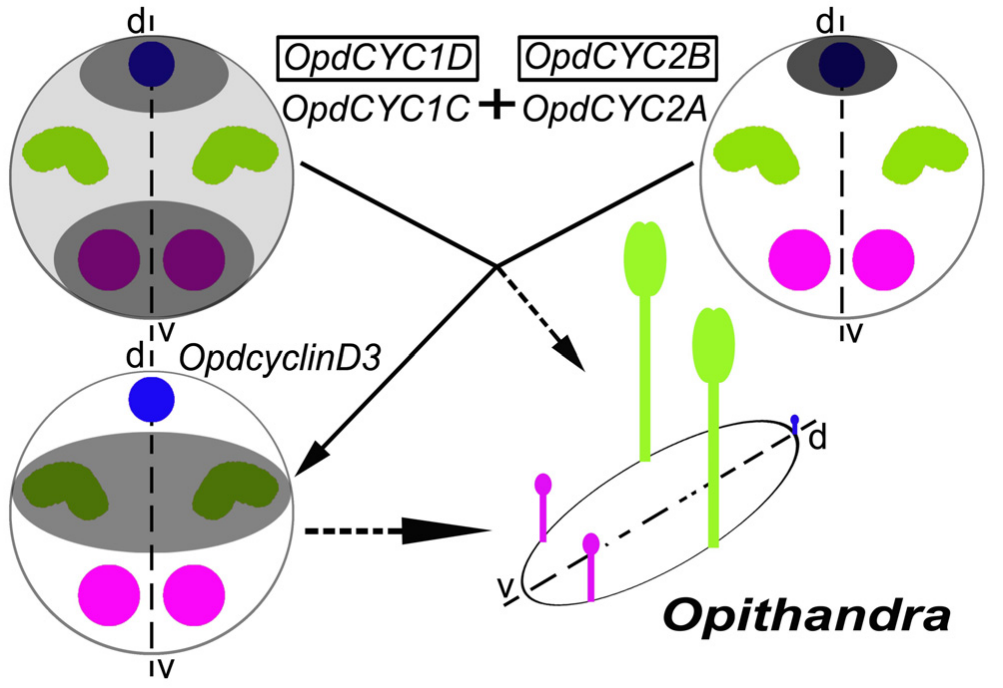
**Diagram showing *OpdCYC *gene expressions correlated with the floral morphology, especially the infertility of the two ventral stamens in *Opithandra*, complemented by the expression of their putative negative target *OpdcyclinD3 *genes**. Notes: dorsal staminode is in blue, lateral stamens in green and ventral staminodes in pink; the gene that is not boxed indicates this gene is expressed in a pattern as shown by the shaded parts within the circle (shaded degrees indicate relative levels of gene expression); the gene boxed indicates this gene is not expressed or has very little expression signals in the floral tissues.

Evidence shows that *CYC *functions to repress stamen development in the third whorl through its negative effect on expression of D3-type *cyclin *genes, including *cyclinD3b*, which usually play an important role in locally regulating cell proliferation in floral development [3,6,36]. The negative effects on cell cycle progression have been reported from other TCP genes, such as *IaTCP1 *from *Iberis *(Brassicaceae), *TCP2 *and *TCP4 *from *Arabidopsis*, and *CIN *from *Antirrhinum *[16,37,38]. We, therefore, investigated the expression pattern of *OpdcyclinD3a *and *D3b *to test for further correlation between *CYC *expression and stamen abortion through cell-cycle regulation, especially the ventral stamens. In strengthening the above suggestion, *OpdcyclinD3 *genes have expression patterns in floral tissues negatively correlated with those of *OpdCYC *genes and stamen abortion in *Opithandra*. Both *Opdcyclin D3a *and *D3b *transcripts are not detected, or weakly detected (i.e. dorsal), in the dorsal and ventral staminodes where both *OpdCYC2A *and *OpdCYC1C *or *OpdCYC1C *are strongly and continuously expressed throughout floral development, while their transcripts are much more concentrated in the lateral stamens where there is only a weak expression of *OpdCYC1C *in early stages. These factors indicate that *OpdCYC *gene activities may suppress the development of the dorsal and ventral stamens through negatively regulating *OpdcyclinD3 *genes (Figure 5). Our recent findings of consensus-binding sites of the TCP transcription factor in the 5' upstream regions of *OpdcyclinD3 *homologues, i.e. *ChcyclinD3a *and *D3b *in *Chirita heterotricha*, further support the direct regulatory relation between *CYC*-like and *cyclinD3 *genes in Gesneriaceae (Yang, Xia and Wang, Yin-Zheng, unpublished).

It seemingly remains a question whether *OpdCYC *activity is related to the lateral stamen development or not because *OpdCYC1C *has a weak expression in the early developing lateral stamens but has no obvious effect on their growth. The evidence that *CYC*-like genes regulate lateral stamen development comes from both functional analyses in *Antirrhinum *and expression data in *Mohavea *and *Chirita *[3,4,21,23,25]. A gradient of *CYC *effect along the dorsoventral axis results in abortion of the dorsal stamen and reduced size of lateral stamens in comparison with ventral ones in *A. majus*, while *CYC*-like gene strong and continuous expressions in both the dorsal and lateral stamens is correlated with abortion of both of them in *Mohavea *and *Chirita *[4,23,25,26]. The *TCP1 *gene is expressed early in floral buds of *Arabidopsis*, but the mature flowers of *Arabidopsis *are actinomorphic because they lack later effects of *TCP1 *[12,15]. In *Iberis amara *closely related to *Arabidopsis*, the *IaTCP1 *strong dorsal-specific expression represses the two dorsal petal growths to become much smaller than the ventral ones in size, while *IaTCP1 *has only a weak expressional signal in the natural actinomorphic variants [16]. In the tetrandrous flowers of *Oreocharis *that is closely related to *Opithandra *in both morphology and *GCYC *phylogeny [24] (Figure 2B), there is also a weak expression of *CYC *homologue *ObCYC *in the early developing lateral stamens that are reduced in size compared with ventral ones at anthesis as in *A. majus *[39]. In *Bournea*, another *Opithandra*'s close relative with actinomorphic flowers in Gesneriaceae, the *CYC *homologue *BlCYC1 *is strongly expressed in the dorsal petal and stamen in early development and is downregulated later, which is correlated with the floral development undergoing a morphological transition from initial zygomorphy to actinomorphy at anthesis with five fertile stamens in *Bournea *[35]. According to Cubas [1], the maintenance of *CYC *expression after early floral development should be important for generating the morphological asymmetries in the flowers. Preston and Hileman [3] also suggest that early expression of *CYC*-like genes may be unimportant for establishing mature flower symmetry. The high concentration of *OpdcyclinD3a *and *D3b *transcripts in the lateral stamens also indicates that *OpdCYC1C *has lost negative effects on their expression after early floral development; therefore, the two lateral stamens are fertile at anthesis in *Opithandra *(Figure 5).

Zygomorphic flowers with three staminodes at the dorsal and lateral positions or at the dorsal and ventral sides have been considered to be derived in the family Gesneriaceae [24,40-42]. In the primitive zygomorphic taxa, such as *Haberlea *and *Oreocharis *characteristic of tetrandrous flowers with four didynamous stamens plus a dorsal staminode, and the derived actinomorphic groups (definition see note in [35]), such as *Ramonda *and *Bournea*, there is only one single copy of *GCYC1 *and *GCYC2*, respectively, found to date (Figure 2B) [35,39,43,44]. However, two copies of *GCYC1 *are frequently found in the advanced zygomorphic taxa, especially in the zygomorphic genera characterized by diandrous flowers with three staminodes, such as two African genera *Streptocarpus *and *Saintpaulia *with *GCYC1A *and *GCYC1B *[44,45] and Asian genera *Didymocarpus*, *Chirita *and *Loxostigma *with *GCYC1C *and *GCYC1D *[25,46] as well as *Opithandra *herein (Figure 2B). Recent studies show that two copies of *GCYC2 *(*GCYC2A/2B*) are also found in the Asian genera with three staminodes, such as *Chirita *[25] and *Opithandra *in this study (Figure 2B). The derived morphology of diandrous flowers might have resulted from subsequent expression differentiation after gene duplication events. In the diandrous flowers of *Chirita *(also Gesneriaceae) that differs from *Opithandra *in abortion of both the dorsal and lateral stamens rather than ventral stamens, *ChCYC1C *is strongly expressed both in the dorsal and lateral stamens while *ChCYC1D *maintains strong expressions in the dorsal floral regions, and *ChCYC2A/2B *have no expression signals in floral tissues [25]. No expression of *GCYC2 *detected in floral tissue is frequently found in Gesneriaceae while *GCYC1 *is usually conserved in dorsal-specific expression in this family, such as *Oreocharis *and *Bournea *[35,39]. Therefore, the peculiar diandrous flowers established in *Opithandra *might involved not only gained or enhanced expression of *OpdCYC1C *in ventral staminodes but also the reactivated expression of *OpdCYC2A *specific to the dorsal staminode accompanied with the downregulation of *OpdCYC1D *in the dorsal region in the third whorl, a more complicated mechanism than that in another diandrous flowers of *Chirita*.

Phylogenetic analyses show that the *CYC*-like genes isolated from *Opithandra *and other Gesneriaceae belong to ECE-CYC2 clade as *CYC *and *TCP1 *from *Antirrhinum *and *Arabidopsis *(Figure 2B) [25,35,39,45]. As outlined above, ECE-CYC2 clade genes are characteristic of dorsal identity function which sometimes expands to lateral stamens [4,5,21,23,25,35,39]. It would be especially interesting to know whether or how *CYC*-like gene activities are related to abortion of the ventral stamens [3,12,20]. Even though not tested functionally, this positive correlation between *CYC*-like gene expression and ventral stamen abortion and the complement of *cyclinD3 *to *CYC*-like gene expressions suggests a genetic mechanism underlying the establishment of zygomorphy with abortion of both the dorsal and ventral stamen evolved within Lamiales *s.l*.. However, it has been shown for *Veronica *and *Gratiola *(also Lamiales *s.l*.) that the *CYC*-like gene expression does not positively correlate with the ventral stamen abortion (Preston et al., 2009). These facts inconsonant in the expression data of ECE-CYC2 clade TCP genes imply that the ventral stamen abortion might have evolved by convergent genetic mechanisms in different lineages of Lamiales *s.l*.. It merits further research in function and upstream regulatory pathway to determine how the expression divergence is caused among paralogues of *OpdCYC *in *Opithandra*. In addition, since the diverse variations of zygomorphy in Lamiales *s.l*. might have involved independent shifts in stamen number [3,21], further investigation of expression pattern and functional analyses of *CYC*-like genes with identification of their upstream *cis*- or *trans*-regulators as well as research in finding other factors possibly coopted to this regulatory pathway in more zygomorphic groups would shed new lights on the mechanisms that underlie the vast morphological diversity of zygomorphy in Lamiales *s.l*..

## Conclusion

As the first to document the expression domain of ECE-CYC clade genes in the ventral stamens, we here report that the expressions of *OpdCYC *genes are correlated with abortion of both dorsal and ventral stamens in *Opithandra*, strengthened by the negatively correlated expression of their putative direct target *OpdcyclinD3 *genes. The complement of *OpdcyclinD3 *to *OpdCYC *gene expressions further indicates that *OpdCYC *expressions are related to the dorsal and ventral stamen abortion through the negative effect on *OpdcyclinD3 *genes. The novel patterns of *CYC*-like gene expressions in *Opithandra*, along with previous reports, suggest that ECE-CYC2 clade TCP genes are not only functionally conserved in the dorsal stamen repression, but also involved in arresting ventral stamens, a genetic mechanism underlying the establishment of zygomorphy with abortion of both the dorsal and ventral stamens evolved within Lamiales *s.l*.. It would be important to further find whether ECE-CYC2 clade TCP genes are recruited repeatedly for arresting ventral stamens and (or) whether there is any other genetic pathways underlying the ventral stamen abortion, independently or interacting with ECE-CYC2 clade TCP genes, in different lineages of Lamiales *s.l*..

## Methods

### Plant materials

All materials used in this study, including gene cloning, *in situ *hybridization and RT-PCR, were collected from the wild fields, i.e. Dinghu Mountains, Guangdong province, China, where plants of *Opithandra dinghushanensis *W. T. Wang are mainly distributed.

### Gene cloning and sequence analyses

*CYC*-like genes were isolated from *O*.*dinghushanensis *using degenerate oligonucleotide primers in 3' and 5'-RACE according to described methods [47] and the manufacture's protocol (INVITROGEN), respectively. Two D3-type *cyclin *genes were also isolated from *O. dinghushanensis *using the above methods. Total RNA was extracted from the floral buds of *O. dinghushanensis *using the Plant RNA Purification Reagent (INVITROGEN) according to the manufacture's protocol. First-strand cDNAs were synthesized from total RNA with the Supertranscript™ III RNase H^- ^Reverse Transcriptase (INVITROGEN). To examine the intron/exon structures we isolated and sequenced the corresponding genomic DNA of *OpdCYC *and *OpdcyclinD3 *genes from leaves. The Oligonucleotide sequences for primers are included in the Additional Material - Additional file 2.

According to the known sequence information, phylogenetic analyses of *CYC*-like and D-type *cyclin *genes were conducted to identify the position of *CYC*-like and D3-type *cyclin *genes isolated herein in their gene families, respectively. AmCYC and AmDICH are from *A. majus *[4,5], and LjCYC1/2/3, TCP1/12/18 and TB1 are from *Lotus japonicus *[13], *Arabidopsis *[12] and maize [34]. GCYC1A/1B are from *Saintpaulia ionantha *and *Streptocarpus primulifolius *[43-45]. GCYC1C/1D (designated as GCYC1 respectively in some taxa) are from *Chirita heterotricha *[25], *Jankaea heldeichii *[43], *Conandron ramondioides*, *Haberlea ferdinandi-coburgii*, *Primulina tabacum*, *Ramonda myconi *[44], *Cyrtandra apiculata*, *Didymocarpus citrinus *and *Loxostigma *sp. [46]. GCYC2 (2A/2B) are from *Chirita heterotricha *[25], *Conandron ramondioides*, *Haberlea ferdinandi-coburgii*, *Ramonda myconi *[44], *Cyrtandra apiculata *[46] and *Didymocarpus citrinus *(Yin-Zheng Wang, unpublished). Amino acid sequences of D-type *cyclin *genes are from *A. majus *(Amcyclin D1/D3a/D3b) [6], *Populus trichocarpa *(Ptcyclin D1/D2/D3) [48], *Nicotiana tabacum *(Ntcyclin D/D3) [49], *Solanum tuberosum *(Stcyclin D) (accession nos.EU325650), *Lycopersicon esculentum *(Lecyclin D3-1) [50] and *Arabidopsis *(Atcyclin D1/D2/D3) [32]. Phylogenetic analyses with the neighbor joining method and p-distance were carried out using PAUP*4.0b4a [51] and bootstrap was estimated with 1,000 resampling replicates. The DNA sequences of genes reported in this paper, i.e. *OpdCYC1C, OpdCYC1D, OpdCYC2A, OpdCYC2B*, *OpdcyclinD3a *and *OpdcyclinD3b *have been deposited in the GenBank database (accession nos. FJ710518, FJ710519, FJ710520, FJ710521, FJ710522 and FJ644637).

### RNA in situ hybridization

Floral tissue for *in situ *hybridization was fixed, sectioned and hybridized to digoxygenin-labeled probes of *OpdCYC1C*, *OpdCYC1D*, *OpdCYC2A *and *OpdCYC2B *with reference to described methods [52]. Four gene-specific fragments of *OpdCYC1C*, *OpdCYC1D*, *OpdCYC2A *and *OpdCYC2B *in the coding region were amplified, respectively, and then were purified and cloned into pGEM^®^-T Easy vectors. Digoxygenin-labeled probes of *OpdCYC1C*, *OpdCYC1D*, *OpdCYC2A *and *OpdCYC2B *were prepared from linearized templates amplified using primer Yt7 and Ysp6 from pGEM^®^-T plasmids [53]. The oligonucleotide sequences for primers are included in the Additional Material - Additional file 2.

### Gene-specific semiquantitative RT-PCR

Flowers of different stages were collected as follows: Flower buds of middle-stage (less than 1 cm long) and flowers of late-stage (3-4 cm long) were collected separately. Sepals were removed from the outer whorl. The petals with corresponding corolla-tube were dissected into dorsal (including the attached dorsal staminode), lateral, and ventral regions. Lateral stamens and ventral staminodes were dissected from the corolla-tube and collected each for RT-PCR. All materials were frozen in liquid nitrogen immediately after collection for ribonucleic acid (RNA) isolation. The extraction of total RNAs, purification of poly (A) mRNAs, and synthesis of the first-strand cDNAs were performed according to the methods described above. The template quantity was regulated to be uniform using the *ACTIN *gene [54]. PCR was performed by using gene-specific primers of *OpdCYC1C*, *OpdCYC1D*, *OpdCYC2A, OpdCYC2B, OpdcyclinD3a *and *OpdcyclinD3b*. To make sure that each pair of primers was suitable, we first used them to amplify genomic DNA of *O. dinghushanensis*. The PCR products were then cloned. At least 20 clones of each PCR product were sequenced, and all the primers used could amplify the specific copies of *OpdCYC *and *OpdcyclinD3 *genes. The following thermocycling conditions were employed: initial denaturation at 96°C for 3 min, 30 cycles of 96°C for 30 s, 55-60°C (depending on the Tm value of primer pairs) for 30s, and 72°C for 1 min, and a final extension at 72°C for 10 min. The amplified products were separated on a 1.5% agarose gel, and the density of ethidium bromide-stained bands was determined using a Bioimaging System (Gene Tools Program, Syngene, UK). We repeated the RT-PCR experiments five times independently with a new RNA extraction each time. In addition, all RT-PCR products were cloned into pGEM-T Easy-vector, and at least 20 clones from each product were sequenced to test the gene specificity of RT-PCR. The *OpdCYC/ACTIN *and *Opdcyclin/ACTIN *ratios represented the relative level of *OpdCYC *and *OpdcyclinD3 *mRNA expression. Data are presented as the mean ± SD of independent RT-PCR experiments, and one-way analysis of variance was used to analyze the expression difference of these transcripts in floral tissue from *O. dinghushanensis*. A P value less than 0.05 was taken to indicate statistical significance. The oligonucleotide sequences for primers are included in the Additional Material - Additional file 2.

## Authors' contributions

CFS and QBL isolated *OpdCYC *and *OpdcyclinD3 *genes and performed the laboratory work of *in situ *hybridization and gene-specific RT-PCR. RHL carried out the phylogenetic analyses of *OpdCYC *and *OpdcyclinD3 *genes. YZW conceived of and designed the studies, and CFS and QBL participated in the design. YZW drafted the manuscript and all the authors participated in the editing of the manuscript. All the authors read and approved the final manuscript.

## Supplementary Material

Additional file 1 **Sequence alignment of OpdCYC and OpdcyclinD3 with other related proteins**. The data provided the sequence alignment of putative proteins encoded by *OpdCYC1C*, *OpdCYC1D*, *OpdCYC2A*, *OpdCYC2B *with AmCYC from *Antirrhinum majus *and *Opdcyclin D3a and OpdcyclinD3b *with AmcyclinD3a and AmcyclinD3b from *A*.*majus*. Click here for file

Additional file 2 **Oligonucleotide sequences for primers used in this study**. The data provided the oligonucleotide sequences for primers used in molecular cloning, RNA *in situ *hybridization and gene-specific semiquantitative RT-PCR. Click here for file
