# Supplementary material for: Expressions of ECE-CYC2 clade genes relating to abortion of both dorsal and ventral stamens in Opithandra (Gesneriaceae)
Source: BMC Evol Biol. 2009 Oct 7;9:244. doi: 10.1186/1471-2148-9-244 (PMC2763874; doi:10.1186/1471-2148-9-244)
Supplement: Additional file 1 — Sequence alignment of OpdCYC and OpdcyclinD3 with other related proteins. The data provided the sequence alignment of putative proteins encoded by OpdCYC1C, OpdCYC1D, OpdCYC2A, OpdCYC2B with AmCYC from Antirrhinum majus and Opdcyclin D3a and OpdcyclinD3b with AmcyclinD3a and AmcyclinD3b from A.majus. [file 1471-2148-9-244-S1.DOC]

**Additional Material**

**Additional file 1** - Sequence alignment of OpdCYC and OpdcyclinD3 with other related proteins.

**A**

**
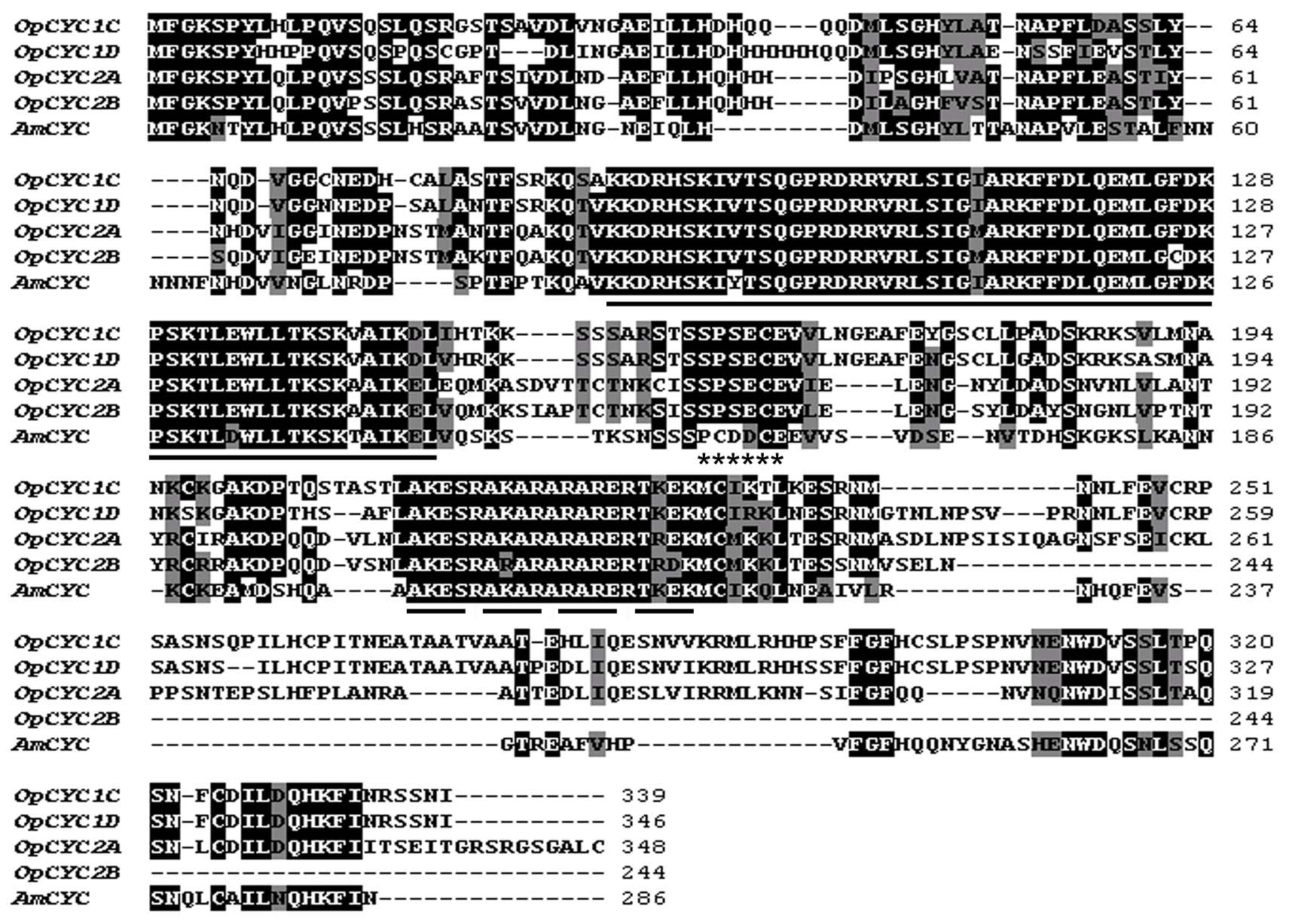
**

**B**


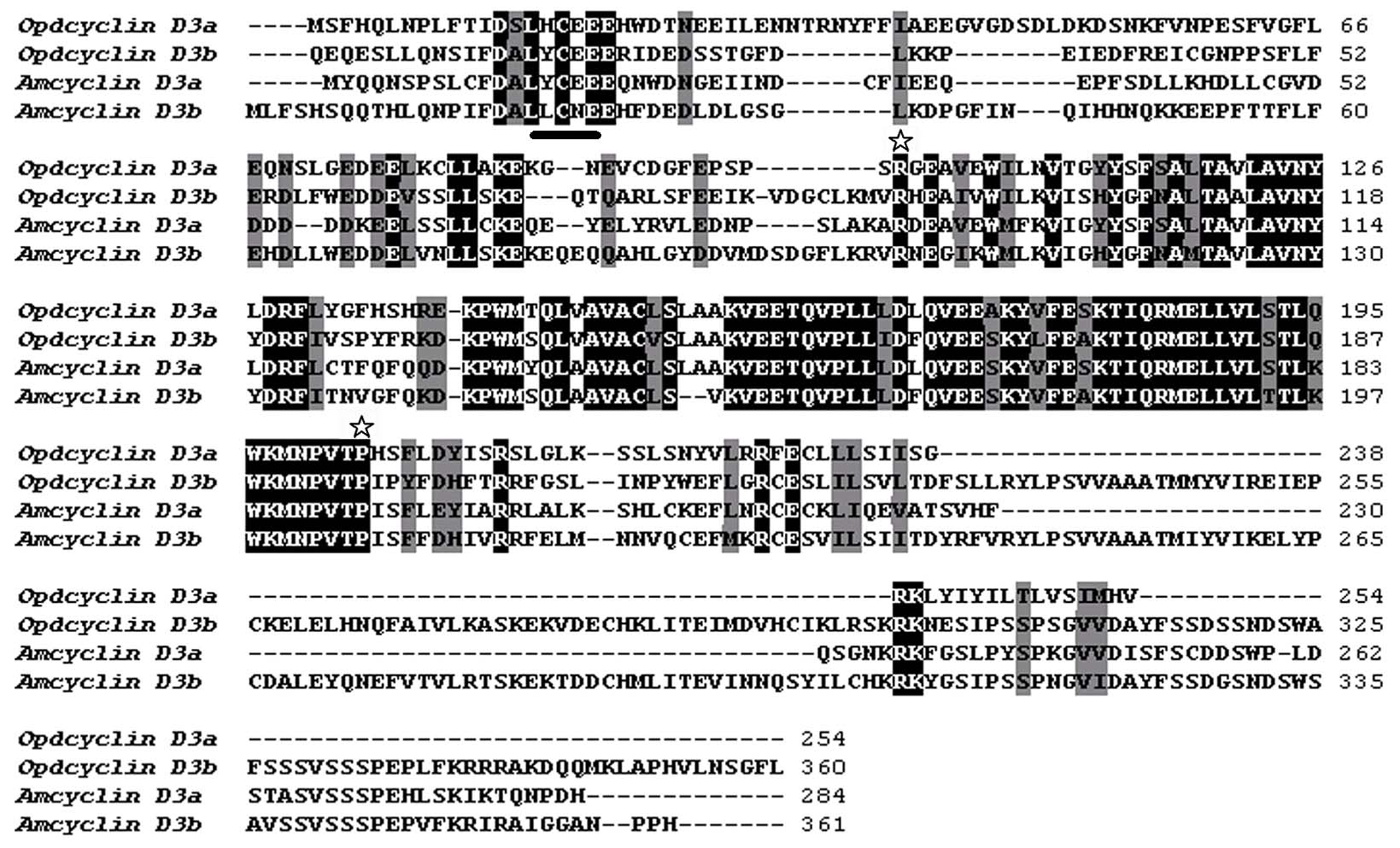


**A)** The alignment of the putative proteins encoded by *OpdCYC1C*, *OpdCYC1D*, *OpdCYC2A* and *OpdCYC2B* with AmCYC from *Antirrhinum majus*. TCP domain is indicated by bold line, ECE motif by stars and R domain underlined with broken line. **B)** The alignment of the putative proteins encoded by *Opdcyclin D3a and OpdcyclinD3b* with AmcyclinD3a and AmcyclinD3b from *A*. *majus*. (Rb)-binding motif（*L*x*C*x*E*）is indicated by bold line and cyclin box is between the two stars.
