# Supplementary material for: Expressions of ECE-CYC2 clade genes relating to abortion of both dorsal and ventral stamens in Opithandra (Gesneriaceae)
Source: BMC Evol Biol. 2009 Oct 7;9:244. doi: 10.1186/1471-2148-9-244 (PMC2763874; doi:10.1186/1471-2148-9-244)
Supplement: Additional file 2 — Oligonucleotide sequences for primers used in this study. The data provided the oligonucleotide sequences for primers used in molecular cloning, RNA in situ hybridization and gene-specific semiquantitative RT-PCR. [file 1471-2148-9-244-S2.DOC]

**Additional Material**

**Additional file 2** -Oligonucleotide sequences for primers used in this study

1. The primers in molecular cloning

3'-fragments of *CYC*-like genes in *Opithandra dinghushanensis* were amplified using oligonucleotide primers FS1 (5'-ATGCTAGGTTTTGACAAGCC-3'), Fcyc1 (5'-TTGTTAACGAAATCGAAAGT AGCC-3'), Fcyc2 (5'-AGTAAAACCCTTGAATGGCTGC-3') and AP (5'-CCGGATCCTCTAGA GCGGCCGC-3') in 3'-RACE. 5'-ORF fragments were amplified using oligonucleotide primers F1 (5'-ATGTTTGGAAAGAGCCCATAC-3'), R (5'-ATGAATTTGTGCTGATCCAAAATG-3') and Rcyc1d-1 (5'-CTCCAGAAGACCTAATTCAAGA-3').

3'-fragments of *Opdcyclin D3a* were amplified using oligonucleotide primers Fd3a (5'-ARTTGGCTGCTGTKGCTTGTC-3'), Fd3a1 (5'-TWGAGGARWCWAAGTATGTGTTTG-3') and AP (5'-CCGGATCCTCTAGAGCGGCCGC- 3') in 3'-RACE. 5'-fragments of *Opdcyclin D3a* were amplified using oligonucleotide primers Rd3b (5'-ATCCAAGACTTCTCGAAATGTAATC-3'), Rd3ab (5'-AATGAAWKTGGGGTSACTGGAT TC-3') in 5'-RACE.

Fd3b2 (5'-TTCAAGAACAAGAATCCCTTCTCC-3') and Rd3b2 (5'-AAGTCCGATCCATCGTCA AAT TG -3') were designed for amplification of *Opdcyclin D3b* from DNA.

PTA (5’-CCG GAT CCT CTA GAG CGG CCG CTT TTT TTT TTT TTT TTT-3’) and AP (5’-CCG GAT CCT CTA GAG CGG CCG C-3’) are universal primers for 3’-RACE.

2. The primers in RNA *in situ* hybridization

Specific to *OpdCYC1C*:

Fcyc1: 5′-TTG TTA ACG AAA TCG AAA GTA GCC-3′

Rcyc1c: 5'-ACA ATG AAG AAT AGG CTG GCT A-3'

Specific to *OpdCYC1D*:

Fcyc1: 5′-TTG TTA ACG AAA TCG AAA GTA GCC-3′

Rcyc1d-2: 5'-GGG ACA ATG AAG AAT ACT ATT AG-3'

Specific to *OpdCYC2A*:

Fcyc2: 5'-AGT AAA ACC CTT GAA TGG CTG C-3'

Rcyc2a: 5'-GGT ATT AGA AGG CGG TAA TTT G-3'

Specific to *OpdCYC2B*:

Fcyc2: 5'-AGT AAA ACC CTT GAA TGG CTG C-3'

Rcyc2b: 5'- CAA GTC AGA AAC GAT GTT G -3'

Universal primers for above probes:

Yt7: 5'-CCC AGT CAC GAC GTT GTA AA-3'

Ysp6: 5'-CAC ACA GGA AAC AGC TAT GAC-3'

3. The primers used in gene-specific semiquantitative RT-PCR

*ACTIN*:

ActinF: 5’-TGT GTT GGA CTC TGG TGA TG-3’

ActinR: 5’-TCC TCC AAT CCA GAC ACT G-3’

Specific to *OpdCYC1C*:

OpdCYC1CF: 5’-ACA AAT GCG CCG TTT CTT GAT GC -3’

OpdCYC1CR: 5’-ACA ATG AAG AAT AGG CTG GCT A-3’

Specific to *OpdCYC1D*:

OpdCYC1DF: 5’-GAA AAT TCG TCA TTT ATT GAG GT -3’

OpdCYC1DR: 5’-GGG ACA ATG AAG AAT ACT ATT AG-3’)

Specific to *OpdCYC2A*:

OpdCYC2AF: 5’-CCT TCG GGC CAC TTA GTA GC-3’

OpdCYC2AR: 5’-CAA GTC AGA AGC CAT GTT T-3’

Specific to *OpdCYC2B*:

OpdCYC2BF: 5’- CTT GCG GGA CAC TTT GTA AG-3’

OpdCYC2BR: 5’-CAA GTC AGA AAC GAT GTT G -3’

Specific to *Opdcyclin D3a*:

Opdcyclin D3aF: 5’- ATG TCT TTT CAT CAA CTA AAC CC-3’

Opdcyclin D3aR: 5’-AAC CAT AGA GAA ACC TAT CCA G-3’

Specific to *Opdcyclin D3b*:

Opdcyclin D3bF: 5’-AGA ACA AGA ATC CCT TCT CC -3’

Opdcyclin D3bR: 5’-AGA GGC ACT TGA GTC TCT TC-3’
